# Complete Desorption of Hybrid Nanoclays Composed of Hydrotalcite and Disperse Dye

**DOI:** 10.3390/ijms241310950

**Published:** 2023-06-30

**Authors:** Daniel López-Rodríguez, Bàrbara Micó-Vicent, Jorge Jordán-Núñez, Ignacio Montava-Seguí, Eva Bou-Belda

**Affiliations:** 1 Departamento de Ingeniería Textil y Papelera, Universitat Politècnica de València, Plaza Ferrándiz y Carbonell s/n, CP 03801 Alcoy, Spain; imontava@txp.upv.es (I.M.-S.); evbobel@upvnet.upv.es (E.B.-B.); 2 Departamento de Ingeniería Gráfica, Universitat Politècnica de València, Plaza Ferrándiz y Carbonell s/n, CP 03801 Alcoy, Spain; barmivi@eio.upv.es (B.M.-V.); jorjornu@eio.upv.es (J.J.-N.)

**Keywords:** nanoclay dyeing, fastness colour dyeing clay, hydrotalcite, dye adsorption, disperse dye recovery, removal of dyes

## Abstract

Clays are considered great nanoadsorbents for many materials, including textile dyes. The use of these materials for cleaning textile wastewater is well known; however, it is not at all common to find applications for the hybrid materials formed from the clay and dye. In this work, a dye-loaded clay material was used to make new dye baths and colour a polyester textile substrate. The same hybrid could be used several times as it did not use all the adsorbed dye in a single dyeing. The hybrid obtained from hydrotalcite (nanoclay) and the dispersed red 1 dye was analysed by measuring the colour obtained, carrying out an X-ray diffraction analysis that provided information after each desorption–dyeing process, and using infrared spectroscopy to analyse the specific bands of each characteristic group. Both analyses showed that the amount of dye present in the hybrid decreases. Thermogravimetry (TGA), surface area and porosity measurements (BET), and X-ray photoelectron spectroscopy (XPS) tests were conducted. Chemical stability was assessed by subjecting the hybrid to the actions of different reagents. In addition, colour fastness tests were carried out after dyeing and washing the polyester test tubes to check for the correct fixing of the dye to the fibre. These fastness results showed that the dyeing was carried out correctly and as if it was a conventional dyeing process.

## 1. Introduction

Cleaning industrial wastewaters by efficiently removing non-biodegradable dyes, which act as pollutants and have a detrimental impact on the environment, is an issue of great concern and a challenge for scientists. The discharge of thousands of tonnes of dyes into water resources, along with other compounds, salts, and bleaching agents, damages the chemical and physical characteristics of aquifers [1]. Among the different physical and chemical methods that can be used to clean industrial wastewaters, the adsorption method is widely employed as it is a cheap, easy, and highly efficient technique for removing organic dyes from water [2]. One of the most widely used nanoadsorbents is hydrotalcite, which is one of the so-called “anionic nanoclays”, a term used to define layered double hydroxides (LDHs) [2].

Due to their ease of preparation, abundance in nature, and ion exchange characteristics [3], LDH clays are among the most widely used and efficient nanoadsorbents. These types of compounds are of increasing interest to the scientific community as an effective environmental substance [4,5] for the removal of dyes. Furthermore, it has been demonstrated that they are capable of adsorbing dyes of any ionic charge and even those that are non-ionic [6], as is the case of disperse dyes.

Disperse dyes are used to dye polyester, nylon, and acetate fibres or blends thereof with other types of fibres; however, but they are not used to dye natural materials such as silk, cotton, or wool, among others [7]. They are not chemically bonded to the fibres [8] but form a kind of alloy when the glass transition temperature of the fibre is reached and the dye can diffuse into the textile structure. Approximately 60% of disperse dyes are azo dyes, which are used to obtain a very wide range of colours. They are cheap and simple to apply and, consequently, are widely used [7].

Several studies have addressed the ability of this mineral to adsorb dyes [9,10,11,12,13,14]; however, in most cases, they have not considered the possibility of desorption and the reuse of the hybrid materials. In the literature, there are cases in which researchers have successfully carried out desorption techniques with laponite [15,16], desorbing 20–40%, as well as using zeolites [17] and sepiolites [18]. Similar to this study, there are studies that have worked with hydrotalcite and with different variables to achieve different degrees of desorption [2]. However, none of these studies have proposed a method in which the reuse of the desorbed material is completed.

The aim of this work is to adsorb a disperse dye using LDH and then collect the clay–dye hybrid and use it to make a dye for polyester fabric (PES). It is expected that several dyes can be made, as the clay will be loaded with a high percentage of disperse dye which will not be completely adsorbed in a single dyeing. Therefore, a battery of dyeings will be carried out in order to study, analyse, and compare the colour intensity obtained and how this process affects the LDH structure.

Some studies [19] have highlighted the use of LDH to stabilise azo dyes, as it is perfectly adapted to their physicochemical characteristics. It should be noted that the incorporation of these new hosts will change their chromophoric properties. Other papers also report optimal conditions on how to synthesise hybrid pigments under the most favourable conditions possible [20,21,22].

In this work, a new method of dyeing through the exhaustion of textile fibres is achieved. Usually, textile dyes are introduced into a dye bath; however, on this occasion, a hybrid nanoclay that has previously adsorbed a dye is introduced instead.

## 2. Results

### 2.1. Dye Adsorption Performance

The dispersed dye red 1 was totally adsorbed by the hydrotalcite [23,24,25,26,27] after the described synthesis process, a result that was expected following previous works by other authors [28]. After reading the absorbance of the filtered water that had fallen due to gravity and calculating the concentration using the Lambert–Beer equation as calculated previously, the adsorption of the dye by the clay was 99.867%.

### 2.2. Hybrid Colour Measurements

**Figure 1:**
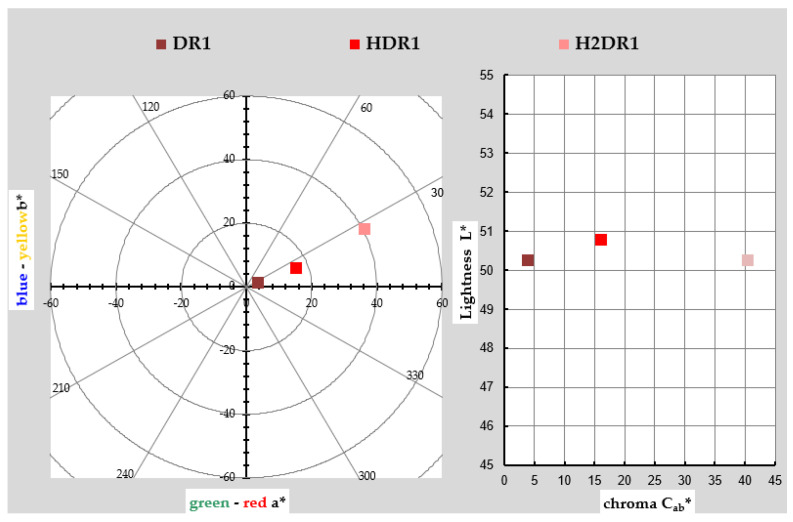
Graphic CIELAB plots for DR1 and hybrid pigments HDR1 and H2DR1 synthesised using the D65 illuminant and the CIE-1931 XYZ standard observer. Left: CIE-a * b * colour diagram; right: CIE-Cab * L * colour chart.

The chromatic coordinates used to represent the colour of the DR1 dye, initial dye-clay substrate (HDR1), and the one collected after the first dyeing (H2DR1) are shown in Table 1 and graphically in a chromatic diagram in Figure 1. To perform these calculations and their corresponding representation, the reflectance values ρ(λ) of each of the samples were taken. The instructions of the CIE 15:2004 standard [29] were used for these calculations along with an objective comparison of the absolute and relative colourimetric results. The illuminant used for the calculations was the standardised illuminant D65 and the CIE 1931 XYZ standard. The comparison of the values of both samples showed that the second H2DR1 lost colour intensity due to desorption on the dye bath and loss of dye charge in the hydrotalcite. When looking at the DR1 dye, the red tones are clearly visible, and they are also visible in the hybrids shown.

However, there are deviations in the shade due to the interaction between the hybrid and the dye. In the hybrid after synthesis, the shade veers slightly toward yellow as a consequence of the white/yellow hue of the nanoclay itself. As expected, this effect increases in the hybrid after dyeing, since the concentration of pure colouring matter decreases in the hybrid after dyeing, and the resulting hybrid, logically, has a shade closer to that of the nanoclay. As far as saturation is concerned, something unexpected occurred: the pure dye sample was the least chromatic, and the intervention of the nanoclay with white and yellowish tones added saturation to the hybrid.

After dyeing, the colour was even more saturated; however, the final result was not as interesting, as this hybrid was far from the initial red tone, veering towards an almost brownish–orange colour, probably due to the thermal degradation of the nanoclay after the dyeing process. This explains why the clarity after the dyeing process (H2DR1) in the hybrid was even lower than that of the original dye, while that of the hybrid after the initial adsorption (HDR1) was higher than that of the original dye, again due to the clarity of the clay that comprises the resulting hybrid.

### 2.3. Measurement of the Colour of Dyes

**Figure 2:**
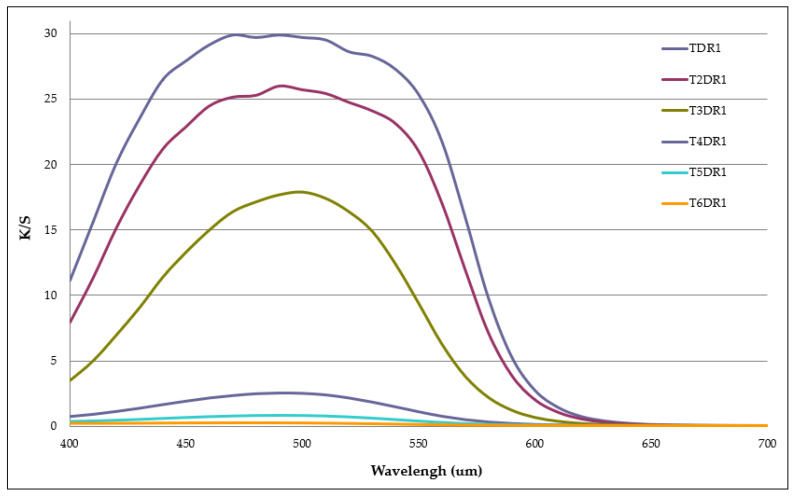
Graphic K/S plots for each dyeing using the D65 illuminant and the CIE-1931 XYZ standard observer.

**Figure 3:**
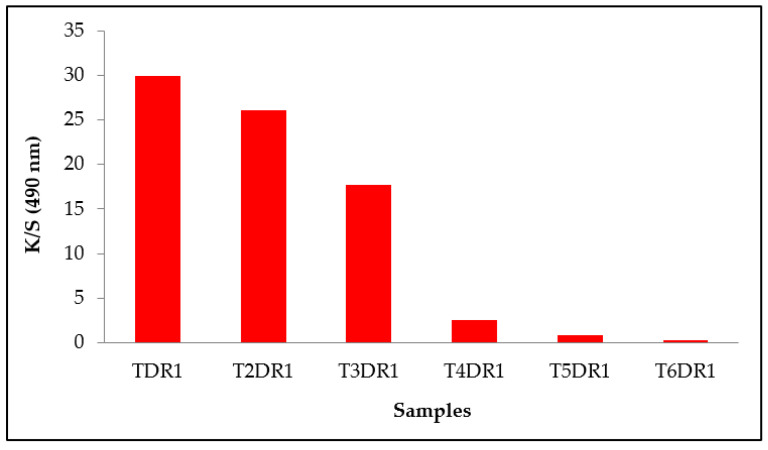
Maximum colour strength K/S at 490 nm for each sample.

The aim of this work was to desorb as much dye as possible that had been adsorbed by the hydrotalcite and to assess the amount of textile material that it was capable of dyeing. For this reason, the evolution of the colour obtained by the fabrics was monitored as the dye cycles were repeated. Table 2 shows the measurements of the values L *, a *, b *, h *_ab_, and C *_ab_, as well as the K/S of each of the six dye cycles that were carried out until the dye in the clay was depleted. The K/S in the range 400–700 nm for each dye is also shown in Figure 2, and the evolution of this colour strength (K/S) is shown in Figure 3 by means of a bar chart.

As there was less and less dye in the clay and all the conditions of the dyeing baths were maintained in terms of bath ratios and the quantity of clay in relation to the weight of the fibre, the results obtained in terms of colour intensity and strength became lower and lower, as was to be expected. The clarity values increased, the saturation decreased, and the tone turned slightly yellowish. There was less dye in the bath desorbed by the clay because, in each of the previous dyeings, it was fixed in the fabric. Therefore, six dye cycles were achieved; however, the last had a very low level of colouring (K/S of 0.25), and it was not necessary to continue with a seventh dyeing.

### 2.4. Colour Fastness

The study of the colour fastness of a coloured textile is a determining factor for the viability of a final product. For this reason, after the dyeing carried out in this work, a colour fastness evaluation was performed for the different agents that could deteriorate it. Clay loaded with a disperse dye that has an affinity, among others, for PES fibres and binds to them when they reach the glass transition temperature as if they were a metallic alloy has been used in previous research [30,31,32]. If the dyeing is carried out correctly, fastness results can be expected to be in accordance with what any conventional dye can offer, with good degrees of fixation in, for example, wet treatments.

#### 2.4.1. Results

Table 3 shows the results obtained for each test and each fabric sample. The values are shown on a scale between 1 and 5, called a greyscale; the higher the value, the more the original colour has been retained. These results can be calculated visually using a greyscale representation or instrumentally by calculating the values with the formulas given in Equation (1a–c). For this study, they were calculated instrumentally. The fastness values increased slightly with successive discharges on the dyes, which is attributable to the fact that the latter dyes have less dye and are therefore less prone to fading.

ΔE_F_ = [(ΔL *)^2^ + (ΔC_f_)^2^ + (ΔH_f_)^2^]^1/2^ (1a)

GS = 5 − [ΔE_F_/1.7] (1b)

GS = 5 − [log_10_ (ΔE_F_/0.85)/log_10_2] (1c)

Equation (1a) Colour change for the determination of the greyscale index for degradation, Equation (1b) GS si ΔE_f_ ≤ 3.4, Equation (1c) GS si ΔE_f_ ≥ 3.4.

#### 2.4.2. ANOVA Colour Fastness

##### Value of Solid as a Function of Sample/Reference

**Figure 4:**
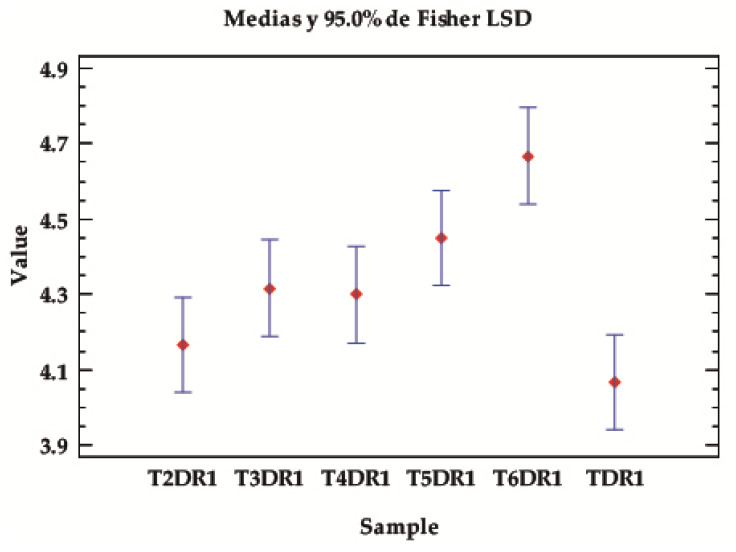
LSD Fisher means plot of the overall average fastness values, according to the type of samples, classified according to the dyeing with the hybrids in different baths, from 1–6 in the established desorption and dyeing stages (TDR1–T6DR1).

In general, depending on the type of sample/reference, there are significant differences in general regardless of the test or conditions, as the *p*-value is almost zero. Table 4 and Figure 4 show how, in almost all cases, the samples from the third dye cycle onwards give higher fastness values than the first cycle, taken as a reference from TDR1, which is only equal to T2DR1. The sample with the highest overall fastness is T6DR1. This is to be expected, since it is the lightest sample with the least colouring. Therefore, the expected degradation is much lower than when there is a higher amount of dye at the beginning. The behaviour is therefore within the expected range. 

##### Value of Solid Depending on Type of Test

**Figure 5:**
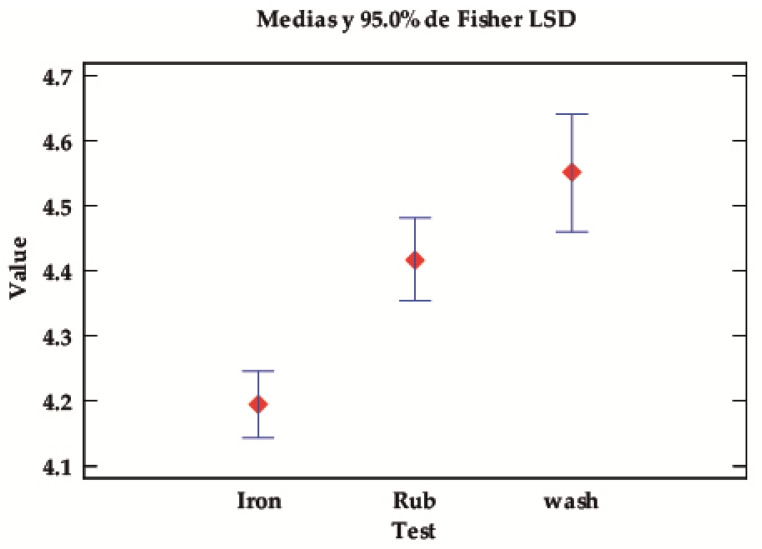
LSD Fisher means plot for the value of the robustness tests.

Depending on the type of test, significant differences can again be seen, with *p*-values below the 0.05 level of significance. Table 5 and Figure 5 show that the fastnesses of this class of pigments are higher in the rub and wash tests and lower in the iron test. In other words, the combination of temperature and pressure affects the properties of the dyes fixed in the fibre more than in the wet and rub tests.

##### Value of Solids Depending on Test Conditions

**Figure 6:**
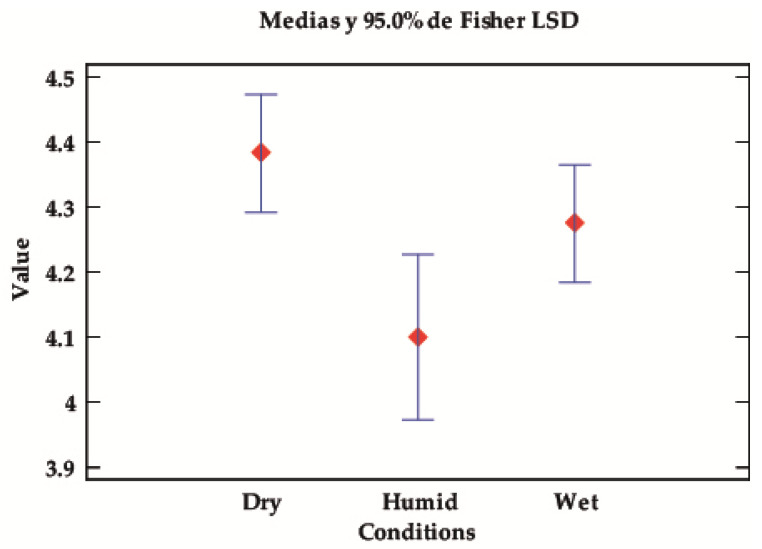
LSD Fisher means plot for the fastness value according to the test conditions (dry, wet, or damp).

Regarding the test conditions, no significant differences can be seen; however, there are trends worth commenting on, as the *p*-values are low and close to the limit of significance. Although there is a slight overlapping of the intervals, it can be observed in Table 6 and Figure 6 that, in dry conditions, the fixed dyes perform better regardless of the moisture/wetness tests.

### 2.5. X-ray Diffraction (XRD)

**Figure 7:**
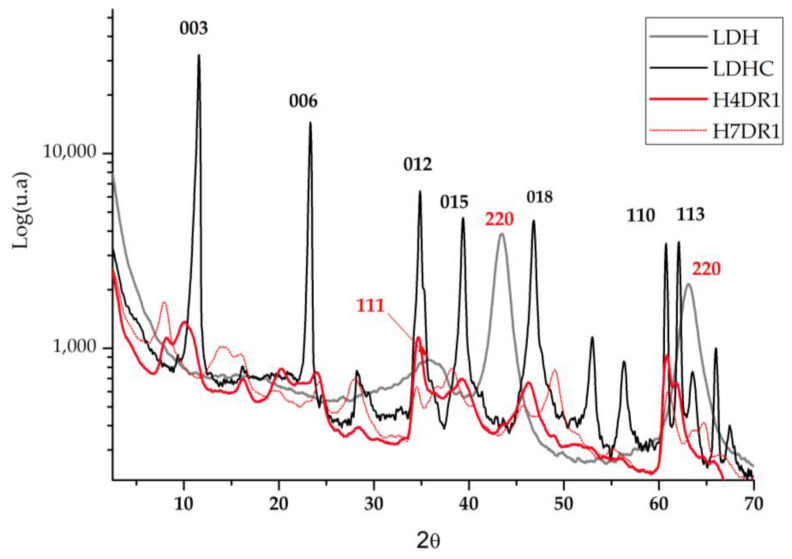
Diffraction patterns of the hydrotalcite without calcining (LDH); the hydrotalcite after the calcination.

Analysing the results obtained from the X-ray diffraction analysis, we compared the hydrotalcite clay before (LDH) and after calcination (LDHC). Figure 7 shows the evolution of the LDH graph, in which different peaks at 11°, 23°, 34°, 34°, 39°, 46°, 60°, and 61° stand out. These are, respectively, attributed to the planes of crystals 003, 006, 012, 015, 018, 110, and 113 [33]. After calcining the nanoadsorbent, the peaks listed above no longer appear, showing new diffraction peaks corresponding to the amorphous mixed oxide structure Mg(Al)O_x_ [34].

In the literature, there are previous analyses based on this work using the XRD technique, in which the Mg-Al elements present the common structuring of hydrotalcite (2θ = 11.27; 34.46°, JCPDS n° 220700). The patterns shown in Figure 7 corroborate these previous works and show the presence of mixed oxides produced by the existence of the Mg(Al^3+^)O of the MgO-periclase type, in agreement with the JCPDS n° 450,946 [35]. Regarding calcined hydrotalcite, we find the corresponding JCPDS n° 211152 due to the MgAl_2_O_4_ spinel structure [36]. After drying the clay again, two phases of the differentiated crystalline structure can be seen: the MgO (JCPDS 450946) phase and the hydrotalcite phase (JCPDS 220700).

When the dye is adsorbed on the previously calcined clay, it can be expected that there will be a slight change in the crystalline structure when the clay is rehydrated and that the dye will affect the diffraction by increasing the amount of amorphous matter. Figure 7 shows how one of the samples used for dyeing H4DR1 had several peaks that were initially in the clay at 23° and 60°, and that one that appeared at 11°, which is shown in detail in Figure 7. However, the H7DR1 sample presents a very different graph to the initial one shown for LDH, which shows how the crystalline structure of the hybrid has changed considerably, as it barely follows the pattern of peaks shown in LDH. These data show that the continuously repeated dyeing process drastically changes the characteristics of the clay.

**Figure 8:**
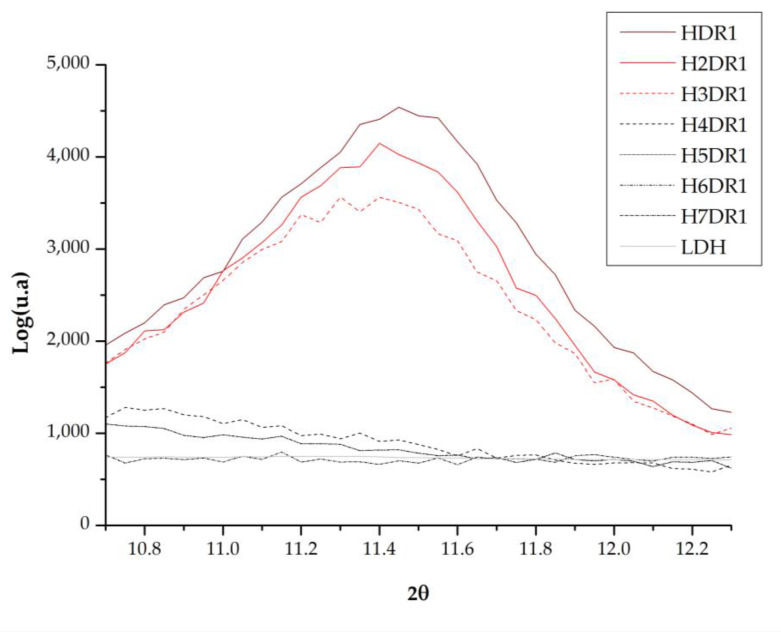
XRD for hydrotalcite calcinated (LDHC); samples HDR1, H2DR1, H3DR1, H4DR1, H5DR1, H6DR1, and H7DR1 in the range of 10° to 12.5°.

A detailed study of the area around 11° in Figure 8 shows that the crystalline structure of the clay is gradually destroyed by the continuous desorption–dyeing process. Thus, H4DR1 shows a collapse of the clay, which practically loses all its crystallinity, leaving a much more amorphous structure [37,38,39].

### 2.6. Fourier Transform Infrared Spectroscopy FTIR-ATR Analysis

**Figure 9:**
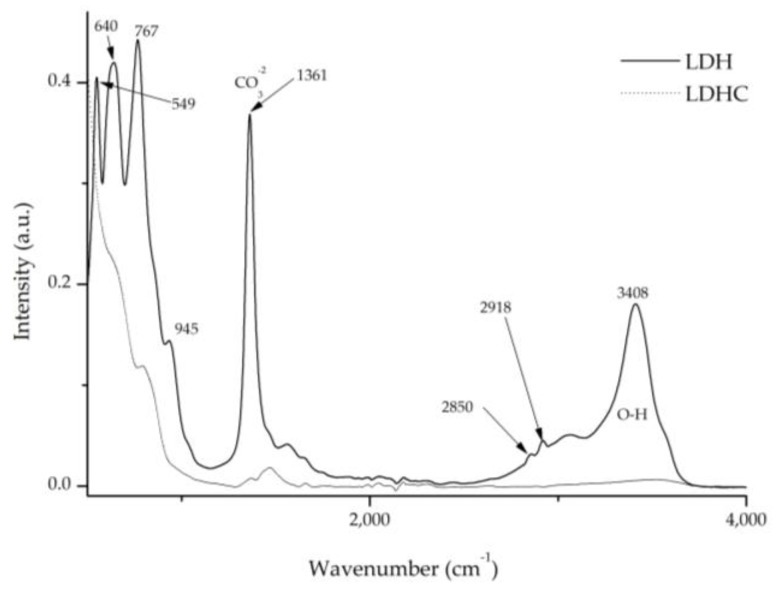
FTIR comparison of uncalcined hydrotalcite (LDH) and calcined hydrotalcite (LDHC).

**Figure 10:**
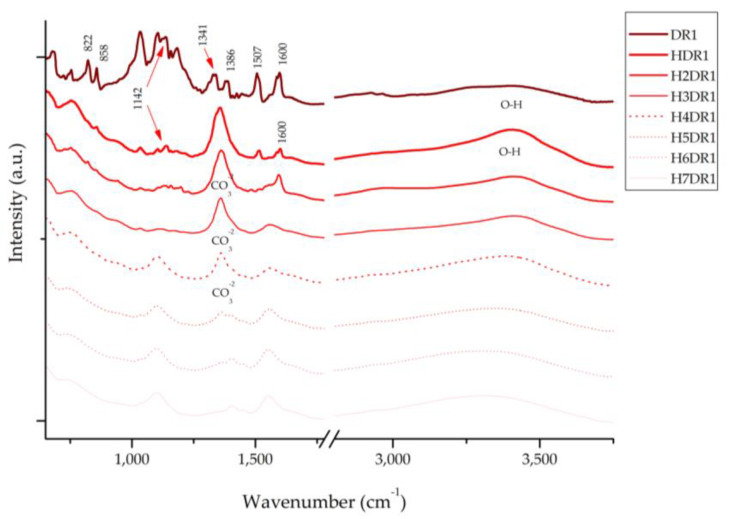
FTIR of dye DR1 and samples HDR1, H2DR1, H3DR1, H4DR1, H5DR1, H6DR1 and H7DR1.

The Fourier transform analysis provides relevant data which can be seen in Figure 9 and Figure 10. Figure 10 shows the differences between calcined and pre-calcined hydrotalcite. There are two prominent bands located at 1360 cm^−1^ and 3200–3600 cm^−1^, which are assigned to the vibrations produced by the carbonate groups -CO_3_^2–^ [40,41] and to the stretching between the oxygen and hydrogen of the hydroxyl group -OH [27,39,41], respectively. Other vibrations that can be located at 2850 and 2918 cm^−1^ are assigned to methylene CH_2_ [42]. Peaks due to the Al–OH bond can also be seen at 766 cm^−1^; moreover, NO_3_- gives rise to another wave at 640 cm^−1^, and the bond between Mg and O produces a vibration at 549 cm^−1^ [39,43].

After the calcination process of the clay (LDH) and the destruction of its structure, all these bands disappear, allowing the nanoadsorbent to incorporate new anionic elements during the reconstruction process, such as dyes with this polarity. These will occupy the place previously occupied by the groups -OH- and -CO_3_^2–^.

Figure 10 presents the spectra of the dye and each of the hybrids collected after the desorption–dyeing process. Upon analysing these spectra, we can see that the characteristic bands of hydrotalcite appear at 1361 cm^−1^, corresponding to CO_3_^2−^, and in the range of 3200–3600 cm^−1^, with the peak centred around 3408 cm^−1^ for the hydroxyl group -OH. It should be noted that these peaks disappear after the calcination process; however, due to shape memory, they reappear during hydration and adsorption. However, these bands attenuate as the dyeing processes continue, which correlates with the results obtained using XRD in Section 2.5, showing, once again, the collapse of the clay as the desorption–dyeing processes continue.

Similarly, the Disperse Red 1 dye has a peak at 1600 cm^−1^ that is produced by the vibrations of the -C=C- bond and two other peaks produced by the asymmetric and symmetric stretching of NO2 located at 1507 and 1341 cm^−1^, respectively [44,45]. Peaks produced by the aliphatic amine groups -C-N-, C-H and the aromatic C-H group, for which the respective peaks are located at 1386, 1142, 858, and 822 cm^−1^, can also be observed [45]. In the first HDR1 hybrid, many of the peaks of the dye are covered by the effect of the clay; moreover, throughout the dyeing process, we can see that the bands are deformed, displaced, and, above all, attenuated—a consequence of the degradation suffered by the hybrid due to the desorption–dyeing process.

### 2.7. BET Surface Area and Porosity Measurements

By means of BET analysis, the surface areas of the clay, as well as the size and depth of the pores, were analysed, all of which are shown in Table 7. A comparative study was carried out between the untreated hydrotalcite samples, both after calcination and after the initial adsorption of the dye. From the results in Table 7, it can be seen that the pore size and depth of the pores increases after calcination, which will facilitate the penetration and adsorption of the dye between lamellae. This is in agreement with other studies, which also indicate that these modifications are due to the degassing of the catalysts produced by the decomposition of the hydrotalcite gases into their hydrated phases [46,47,48]. A reanalysis of the clay after the adsorption of the dye and reconstruction of the structure via rehydration shows very similar values to those initially presented.

### 2.8. X-ray Photoelectron Spectroscopy (XPS)

**Figure 11:**
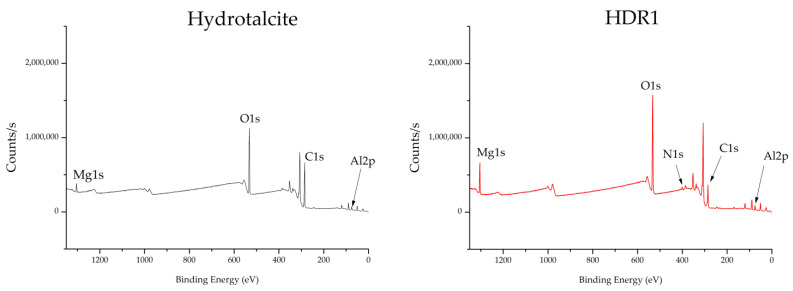
XPS hydrotalcite and HDR1.

The XPS analysis of the hydrotalcite and the HDR1 hybrid is shown in the graphs in Figure 11. In addition, Table 8 presents detailed information on the bond energy values for a number of atoms selected as the most relevant for testing the presence of the dye and the clay. Thus, the presence of C1s, O1s, Al2p3, and Mg1s is observed in various peaks at 285, 530, 174, and 1308 eV, respectively. The presence of N1s is also demonstrated by the peak at 399–401 eV in the HDR1 sample, a determining element for the Disperse Red 1 dye.

The XPS results are complemented by the FTIR results, thus reinforcing the demonstration of the presence of the characteristic elements of the dye and also of the nanoclay. The elements are only present in the hydrotalcite that converted their percentage presence in the hybrid; therefore, the structure of the clay did not undergo noticeable variations and retained its characteristics in terms of thermal and chemical resistance. 

X-ray photoelectron spectroscopy (XPS) was used to corroborate the presence of Mg, Al, and N. Figure 11 shows the binding energy spectra corresponding to the Mg1s and Al2p3 orbitals. The maximum peak of the XPS Mg1s signals of all analysed samples was located at a binding energy of 1308.68 eV for LDH and 1303.82 eV for HDR1. The respective positions of the XPS Mg1s lines are attributed to magnesium compounds such as MgCO and Mg(OH)_2_ [49].

The maximum XPS Al2p3 peak for all the materials examined was found at a binding energy of 73.82 eV. A binding energy close to 74 eV for the Al2p3 line is attributed to a number of aluminium compounds with Al–O bonds, such as various polymorphs of Al_2_O_3_, AlOOH, or Al(OH)_3_ [50]. The chemical shifts reported for these compounds are minor, and phase identification based on binding energy alone can be misleading. However, Al 2p lines attributed to Al_2_O_3_-type bonds are generally above 74 eV, whereas aluminium compounds with -OH groups have XPS Al 2p lines located below 74 eV. After modification with Alizarin, the Al 2p maximum was not shifted.

### 2.9. Thermogravimetry (TGA)

**Figure 12:**
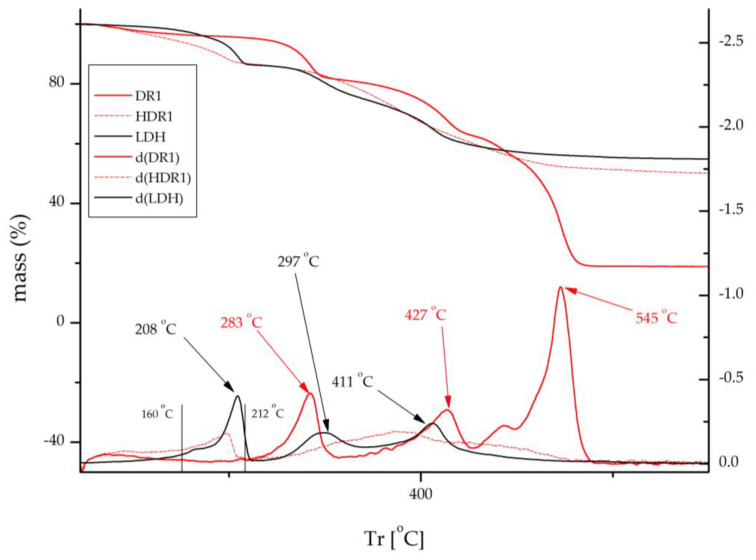
TGA and DTGA dye Disperse Red 1 (DR1), hydrotalcite (LDH), and sample HDR1.

Figure 12 shows the results obtained after TGA and DTGA thermogravimetric analysis. The upper part shows the percentage of mass that the hybrid loses due to the effect of heat, while the lower part of the graph shows the curve d(DR1), which corresponds to the derivative of this loss. Above 500 °C, the dispersed dye suffers an abrupt drop, resulting in a loss of mass of more than 80%. This shows that this type of dye has a low fastness to energetic factors such as ironing or light. 

The figure compares the behaviour of LDH, the dye DR1, and the hybrid formed by both. For LDH, a mass loss due to the evaporation of interlaminar H_2_O can be seen at temperatures between 100 and 170 °C. Due to the loss of the lamellar hydroxyl -OH groups, there is another drop in the range 172–279 °C. A final mass drop is seen in the range 275–605 °C, which can be attributed to the loss of CO_3_^2−^ [40,51,52].

In the case of DR1, three distinct temperature peaks can be observed at 283, 427, and 545 °C. The peak at 545 °C is the most intense and significant, as it indicates a strong degradation of the dye at high temperatures and is accompanied by a considerable mass loss compared to the other dyes. However, after the clay adsorbs the dye, these three peaks disappear completely in the HDR1 sample. Instead, a smooth rise is observed in the range of 160–212 °C, which is characteristic of clay, and is similarly present in the other samples.

Several expert investigations have revealed that the effect of hydrotalcite has contributed significantly to an increase in thermal stability in dye–clay hybrids [53,54]. This is due to two main factors: firstly, the lamellar structure of the nanoclay acts as a protective agent that decreases the volatilisation of the dye components; secondly, when the composite is subjected to high temperatures, an energy transfer from the dye to the clay occurs. This transferred energy is absorbed by the nanoclay, which reduces the impact of energy on the dye [55,56].

### 2.10. Resistance to Chemical Attacks (Chemical Stability)

To evaluate the resistance of the hybrid to different chemical agents, the HDR1 sample was subjected to a temperature of either 150 or 200 °C to perform an initial weakening of the bonds and then subjected to the action of acidic and basic solvents. Table 9 shows a design of experiments where the different tests proposed were carried out, combining the variables. 

### 2.11. Morphology Scanning and Transmission Electron Microscopy (SEM-TEM)

**Figure 13:**
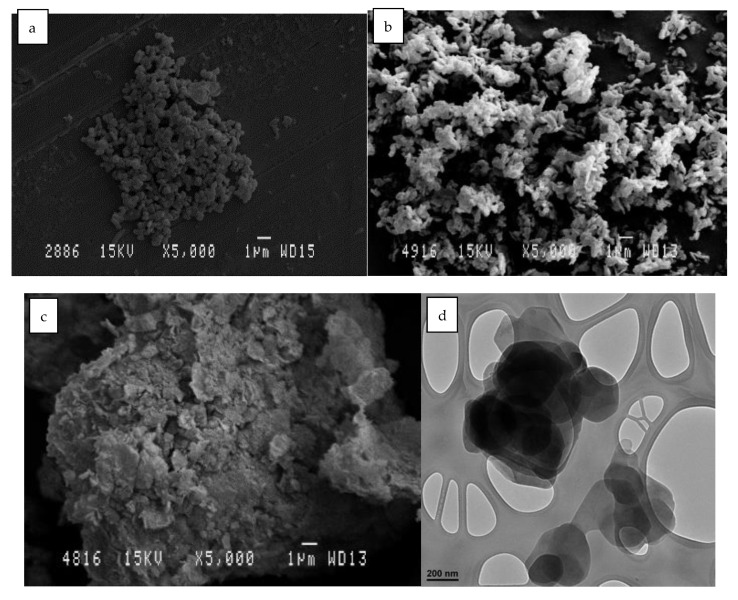

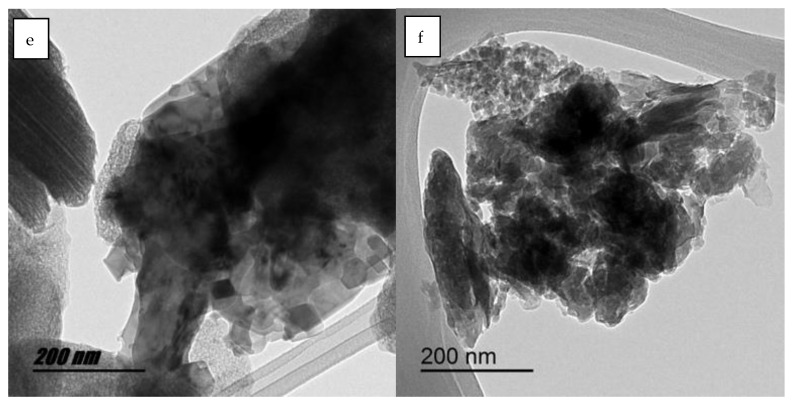
SEM micrographs of different LDH samples: (**a**) LDH original; (**b**) HLDH calcinated; (**c**) LDH reconstructed. TEM micrographs of different HLDH samples: (**d**) LDH original; (**e**) LDH calcinated; (**f**) LDH reconstructed.

SEM-TEM photographs are shown in Figure 13. In these images, the difference before and after calcination can be seen. The morphological surface shows that, after the rehydration and the recovery of the structure, it resembles the initial structure. It should be remembered that this reconstruction is due to the shape memory that this clay possesses, which has already been verified with the XRD results.

In Figure 13a,d, we can clearly see crystals with the characteristic hydrotaltic structure. After calcination Figure 13b, the crystallinity of the clay was lost, while the agglutination was increased in Figure 13e. Finally, after rehydration and adsorption of the dye, in Figure 13c, it can be seen that the crystalline structure was reconstructed, and the TEM image in Figure 13f shows an intermediate agglutination size between the two previous phases (before and after calcination).

## 3. Discussion

After adsorbing the non-ionic disperse dye using the hydrotalcite nanoadsorbent, several dyes were successfully produced with this hybrid. To achieve this, the opposite process to that of dye adsorption was carried out. This desorption had a sufficiently efficient performance to produce dyes of high tonality and chroma, giving rise to coloured polyester textiles as if they were conventional dyes. This desorption and dyeing process was achieved by collecting the clay after each dyeing and effectively reusing it in a new bath. Obviously, the K/S colour strength of the fabrics was reduced after each dyeing, as the concentration of dye remaining in the clay decreased. 

The dyed substrates were subjected to colour fastness tests to check the correct binding of the dye to the fibre, and these led to excellent results in accordance with the characteristics and values that a common dyeing of polyester fabrics with disperse dyes can offer.

The adsorbent capacity of hydrotalcite was once again confirmed in this study. On this occasion, the dye adsorbed was non-ionic and is capable of adsorbing dye by processes other than ion exchange. The FTIR and XRD analyses show how LDH is able to rehydrate and recover its structure due to its shape memory and the presence of the dye in these hybrids. This is evidenced by the presence of differentiating bands of amino or sulphonate groups in FTIR and N after XPS and EDX analysis. These analyses also show how the nanoclay deteriorates as the dyeing process is repeated and desorption occurs.

## 4. Materials and Methods

### 4.1. Materials

**Figure 14:**
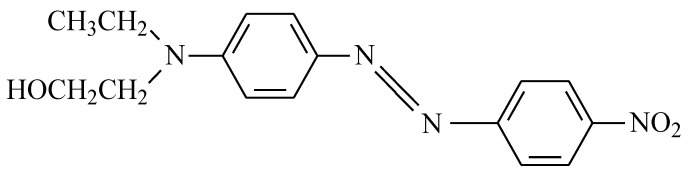
Chemical structure of the non-ionic dye Disperse Red 1.

In this work, we used a non-ionic dye, without polarity, of the disperse class, referenced as Disperse Red 1 CI 11,110 (DR1), supplied by the company Clariant, whose chemical structure is shown in Figure 14. As it is a non-ionic dye, ion exchange techniques cannot be used for its adsorption; however, it is expected that this will contribute to a more efficient and simple desorption. This dye is commercially distributed with a certain amount of dispersing agent to facilitate its dispersion in water, as it has low solubility [57]. It should be noted that, due to the objectives of this work, this dye has high ecotoxicity both before and after chlorination [58,59].

**Figure 15:**
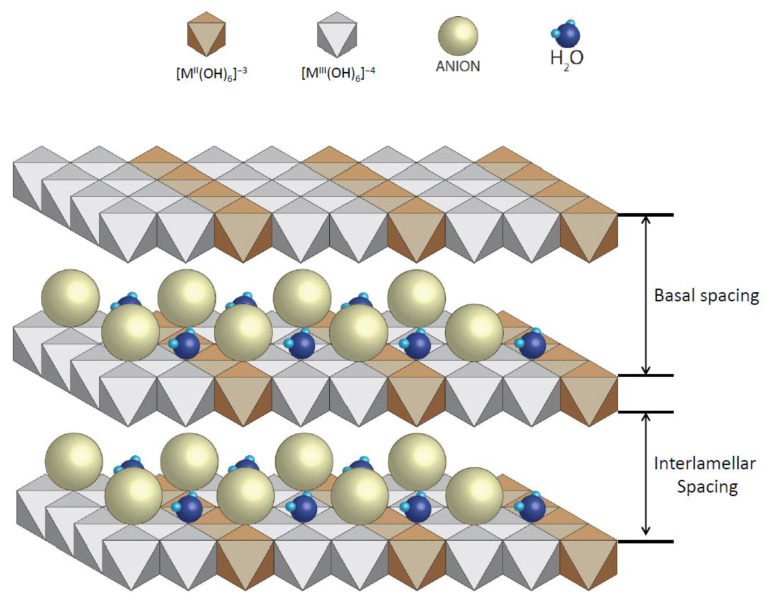
Basic structure of hydrotalcite.

In order to adsorb the dispersed dye, it was necessary to use a nanoadsorbent. In this work, we used clay from the LDH type, specifically, hydrotalcite: Mg_6_Al_2_(CO_3_)(OH)_16_·4(H_2_O) [60,61,62]. Due to the fact that the chosen dye is non-ionic, it was possible to work with LDH without any previous preparation to increase its ion exchange capacity; however, we decided to prepare it following the work of Dos Santos R.M.M. [27] in order to compare results with other works and future research. This preparation consisted of introducing the clay to an oven at 600 °C for 3 h to destroy its structure, which was then reconstructed thanks to its shape memory when it comes into contact with water in the dye adsorption phase. Figure 15 shows a representation of the structure of hydrotalcite and shows the basal and interlamellar spacing of its lamellae.

### 4.2. Synthesis Methods

Wastewater from a conventional dyeing process contains dyes that have not been absorbed by the fibre during the dyeing process, and which are going to be discharged or subjected to a purification process. In this work, clay was used to adsorb the dye in solution or aqueous dispersion. To control both the amount of dye in the dye bath and the amount that may remain after adsorption, it was necessary to calculate the Lambert–Beer line [58]. By making several dilutions of the dye at certain known concentrations, it was possible to obtain this line with its corresponding equation (Equation (1a–c)) to calculate the amount of dye that would be present in each case.

Y = 25.411x − 0.0244; R^2^ = 0.9989 (2)

Equation (2) Lambert–Beer line equation and R^2^ for Disperse Red 1 (DR1).

Adsorption studies were carried out with real dye wastewater, and the effectiveness of the adsorption method was proven. However, certain conditions were simulated in the laboratory, which are detailed below [63,64]. Five litres of dye bath were prepared with only the dye, without adding any type of salt, dispersant, or any other product, since the initial objective was to load the clay with only the dye. These 5 litres had a concentration of 1 g·L^−1^ of dye, to which, once the dye was dispersed, 3 g·L^−1^ of hydrotalcite was added. The dispersion was then stirred to maximise dye adsorption. This agitation was carried out with a magnetic stirrer and consisted of two phases. In the first phase, a stirring speed of 1600 r.p.m. was applied for 2 h. In the second phase, the stirring speed was reduced to 500 r.p.m. for a further 22 h, so that the adsorbed dye did not escape from the clay due to overly energetic stirring [65]. 

The thermogravimetric analyser TGA/SDTA 851 (Mettler-Toledo Inc., Columbus, OH, USA) was used to evaluate the thermal resistance of the dye, LDH, and hybrids using thermogravimetric analysis (TGA). For this experimental process, a temperature increase rate of 5 °C every minute was used for a range from 20 to 900 °C in an oxidation medium of N2:O2 (4:1).

After achieving the adsorption of the dye, the hybrid obtained was separated from the water, for which a filtration process was carried out so that all the water passes through by gravity and the clay–dye hybrid is retained by the filter. At this stage, the solid phase remains in the upper part, while the liquid part falls. This process was carried out for 48 h to ensure that all the water passed through a 130 g·m^−2^ filter paper with pores of 25–30 µm and a thickness of 430 µm. The collected water was analysed in an absorption spectrophotometer to calculate the amount of dye remaining in the simulated wastewater [66,67]. At the same time, the solid hybrid was dried at 70 °C in an air oven to eliminate any remaining aqueous residue, obtaining the hybrid (HDR1) resulting from the adsorption of the Red Disperse Dye 1 and the hydrotalcite.

### 4.3. Dyeing Method and Desorption

The reverse process in which the dye leaves the clay and returns, for example, to an aqueous phase, is called desorption [68,69]. Different models by various researchers [70,71,72] have offered different ways of achieving this desorption process. Some of them are described as continuous adsorption/desorption models, in which the system is non-ideal and can be reversed. Some also describe them as systems in which the adsorption is not completely equal and homogeneous; therefore, there is no clear interaction between all the active groups; furthermore, they are not sufficiently fixed to the clay structure. Momina, Shahadat Mohammad, and Suzylawati Isamil [73] proposed a model in which it is essential to use two phases to obtain good results, as either phase alone is not sufficient. One phase is based on subjecting the hybrids to high temperatures to weaken the bond between the dye and clay molecules, and then finishing the separation by immersing the compound in substances that break the adsorption, such as nitric acid, hydrochloric acid, or acetone [73].

**Figure 16:**
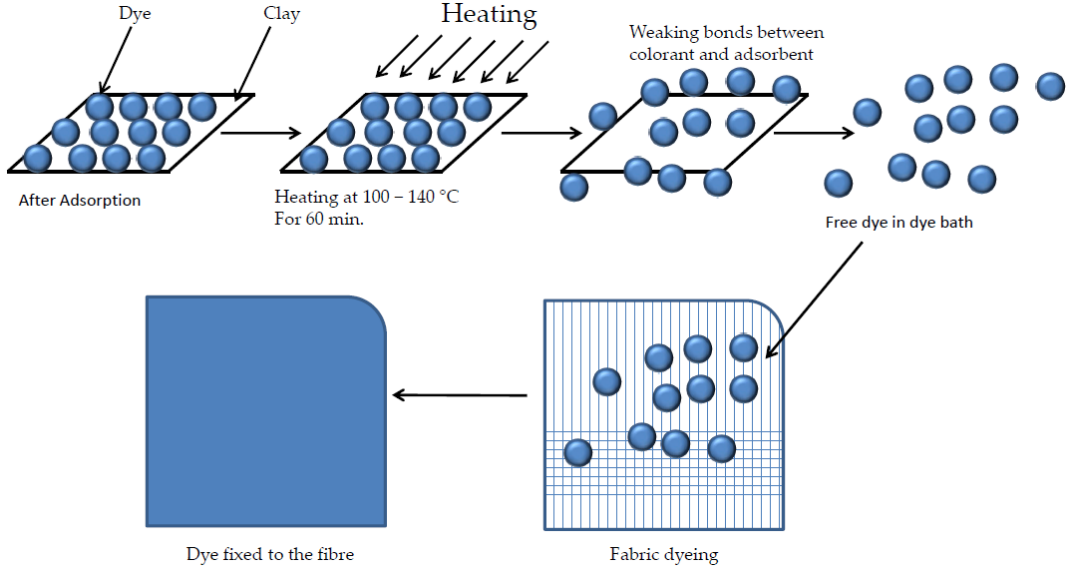
Disperse dye desorption and dyeing PES.

After studying other research, we decided to apply a different technique for this study based on applying convective heat such as that of a bath at 130 °C and, at the same, time introducing all the additional elements necessary (dispersants, salts, etc.) to carry out a PES dyeing (Figure 16). In this way, the energy introduced is used much more effective when performing the separation; moreover, by performing the dye–fibre bonding at the same time, it is not possible for the dye to be re-adsorbed by the clay and produce a barrier effect to successfully complete the desorption.

In accordance with the above, the clay loaded with HDR1 dye was used as an element to provide the colour to the textile substrate in a closed machine dyeing process by exhaustion. Obviously, the clay itself cannot provide any colour; however, in the desorption process, the dye that is trapped by the hydrotalcite is released from the hybrid, remaining in dispersion in the bath, and allowing the dyeing process to take place. The substrate to be dyed was a 200 g·m^−2^ 100% PES plain weave calada fabric with densities of 13 yarns·cm^−1^, 52 weft·cm^−1^. 

The bath ratio to used was 1/40, and the amount of hybrid was 40 on fibre weight. This bath was adjusted to a pH of 5, and 1 g·L^−1^ ammonium sulphate (NH4)_2_SO_4_ and 0.5 g·L^−1^ Dekol SN dispersant were introduced to achieve a good dispersion of the dye, which is not soluble. The dye bath with all the elements listed was subjected to a heating process at 130 °C for 30 min. As the temperature is higher than 100 °C, in order to avoid evaporation, it must take place in a closed dyeing machine (Testherm type 9S from the manufacturer Talcatex S.A., San Sebastian de los Reyes, Spain). After dyeing, the fabric was washed with distilled water, and this water and the remaining water in the dye bath was collected and filtered again using gravity, as in the synthesis of the hybrid; then, the remaining clay was collected. The rest of the fabric was washed at 60 °C for 30 min with non-ionic detergent and a dispersant-sequestering agent to remove the dye residues that remained fixed to the fibre. This process was repeated with the clays recovered after each dyeing until a total of 6 cycles were carried out, at the end of which there was only a very slight dyeing of the fabric, and the clay was considered to be exhausted for dyeing processes.

### 4.4. Characterisation

A Jasco V-670 double UV–VIS/NIR spectrophotometer was used to calculate the colour of all the hybrids, both initial, inter-meridian, and final, working in the range between 190 and 2700 nm and measuring with a frequency of 0.5 nm between each measurement. This was equipped with a monochromator with a double grating for each region, the two regions being the UV–VIS and the infrared IR. The two gratings had an autonomous system capable of detecting the wavelength at which one is working and making use of each of them according to the needs of the analysis. The CIE-1964 observer and the D65 illuminant were used, applying the reflectance factors. A halogen lamp (330–2700 nm) and a deuterium lamp (190–350 nm) were used as illumination sources [74].

A scanning electron microscope (SEM) model PHENOM (FEI Company, Eindhoven, the Netherlands), operated at an acceleration of 5 kV, was used to visually analyse the topography of the dyed tissues. In order to have a conductive material, the samples were prepared in advance using a spray coating of a conductive alloy composed of palladium and gold. This coating was carried out with an EMITECH sputtering machine mod. SC7620 (Quorum Technologies Ltd., East Sussex, UK). Preparation with the coating will not alter the imaging result, as the thickness is less than 10 nm.

The different clays were loaded with dye and, after their respective desorptions, were analysed using an infrared spectrophotometer. Given the physical characteristics of the element to be analysed, an analysis was carried out using the horizontal attenuated total reflection technique (FTIR-ATR), with the use of a ZnSe prism. For the reading, a Jasco FTIR 4700 IRT 5200 spectrophotometer with a DTGS detector sensor was used. The use of a pressure accessory was necessary to obtain uniform readings for all samples. 

For the study of clay crystallinity and the influence of dye adsorption/desorption, an X-ray diffraction (XRD) analysis [75,76] was carried out to analyse and compare the behaviour of each sample. Special attention was paid to the basal space between the clay sheets, which can alter the levels and capacities of dye adsorption. We worked in an oxidising atmosphere with an angular velocity of 1°/min, STEP 0.05°, and an angular sweep of 2.7–70° and used the RD bruker D8 Advance equipment (Bruker, Billerica, MA, USA), with a Göebel mirror (power: 3000 W, voltage: 20–60 kV and current: 5–80 mA).

For the measurement of the colour of each of the dyed fabrics, a Minolta CM-3600d reflection spectrophotometer and Spectramagic software were used, along with D65 illuminant. An observation at 10° with specular radiation was excluded. The same software was used to calculate the chromatic coordinates, K/S and Eab*, from the reflection results.

All the dyeing samples were assessed for colour fastness to check the correct fixing of the dye to the fibre. The colour fastness to washing was carried out following the instructions of the UNE-EN ISO 105-C06:1994 standard using the Linitest according to this standard. Depending on the needs of each product, tests with different characteristics can be carried out. In this case, the A1S test was selected, in which the washing was carried out in a 150 mL bath at 40 °C for 30 min. No pH adjustment was made and, in order to generate a more abrasive action, 10 standardised steel balls were added. Ironing fastness tests were also carried out on wet, damp, and dry ironing according to the UNE-EN ISO 105-X11 standard, using a pressure plate. These ironing tests were carried out at a temperature of 200 °C for 15 s. Finally, dry and wet rubbing colour fastness tests were carried out using a crockmeter and the indications given in the UNE-EN ISO 105-X12 standard.

The fastness results were expressed on a scale of 1 to 5 according to the greyscale. These results can be assessed visually in comparison with a greyscale or, as in the case of this work, they can be measured using equipment, and the result can be calculated mathematically and expressed according to the greyscale. The corresponding reflection measurements were carried out with the Minolta CM-3600d reflection spectrophotometer, as in the colour analysis, and the UNE-EN ISO 105-A05 standard was followed to calculate the discharge and colour degradation indices of the samples.

In order to determine the surface area, pore volume, and pore size, a BET analysis was carried out using different nitrogen adsorption and desorption values at −196 °C using Micromeritics ASAP-2020. The samples were initially degassed in a controlled vacuum atmosphere at temperatures in the range of 140 °C to 200 °C to decompose any sensitive atoms in the analysed samples [47,48].

For the energetic study of the bonds, X-ray photoelectron spectroscopy (XPS, NEXSA, Thermo Scientific) was used for surface analysis using radiation (1486.6 eV), monochromatised by a double crystal monochromator, which forms a focused X-ray spot 400 µm in diameter, at 6 mA × 12 kV. The equipment was equipped with an alpha hemispherical analyser, which was operated in constant energy mode with analysis step energies of 200 eV for full energy band measurements and 50 eV in a narrow scan to obtain the results of individual elements. Thus, XPS was used to obtain the chemical bonding state and elemental composition of the surface of the samples. Charge compensation was achieved with the flood gun system, whereby low-energy electrons and low-energy argon ions were obtained from a single source.

The experimental model published by Momina, Shahadat Mohammad, and Suzylawati Isamil [73] provides a methodology with which to evaluate the chemical resistance using methylene blue (MB) desorption. This method is based on subjecting the clay–dye hybrid to different temperature conditions and using solvents such as HCl and NaOH. The experimental process begins by heating the sample of the dry, solid hybrid collected in the experimental adsorption step at approximately 150–200 °C for 45 min. This weakens the bonds between the adsorbent and the dye. In the next step, HCl or NaOH is added where appropriate to the experimental sample, and the mixture is stirred for 30 min. Finally, the mixture is filtered to separate the clay solids from the solution.

## 5. Conclusions

This work demonstrated the capacity of hydrotalcite to adsorb non-ionic textile dyes of the disperse type and to carry out a complete desorption process through a battery of dyes until a complete desorption of all the clay was achieved. Thanks to this process, six dyes were produced in a controlled way. The colour strength of each of these dyes was measured by means of the K/S calculation, and it was observed that, after each dyeing and collection of the residual clay, the following dyeing had a lower intensity than the previous one, obtaining a diverse range of colours.

FTIR, XPS, SEM, and XRD characterisation tests have demonstrated the destruction of the nanoclay structure and its subsequent recovery when introduced into the aqueous phase. In addition to the presence of both the clay and the dye in the hybrid obtained, its temperature resistance was tested using TGA analysis, showing the thermal protection provided by the clay to the dye.

## Figures and Tables

**Table 1:** Values L *, a *, b *, C *_ab_, and h *_ab_ of each hybrid.

Sample	L *	a *	b *	C *_ab_	h *_ab_
DR1	50.26 ± 0.72	3.59 ± 0.23	1.3 ± 0.33	3.82 ± 0.15	19.84 ± 0.02
HDR1	50.77 ± 0.52	15.14 ± 0.61	5.86 ± 0.23	16.24 ± 0.52	21.17 ± 0.74
H2DR1	50.89 ± 0.08	12.73 ± 0.72	3.45 ± 0.12	13.19 ± 0.23	15.16 ± 0.36

**Table 2:** Values L *, a *, b *, C *_ab_, h *_ab_ and K/S of each dyeing.

Sample	Dye Shade	L *	a *	b *	C *_ab_	h *_ab_	K/S (490 nm)
TDR1	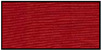	32.43 ± 0.43	50.26 ± 0.15	30.71 ± 0.11	58.90 ± 0.69	31.43 ± 0.32	29.87 ± 0.98
T2DR1	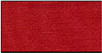	35.04 ± 0.29	51.61 ± 0.11	31.56 ± 0.97	60.50 ± 0.09	31.45 ± 0.32	26.03 ± 0.77
T3DR1	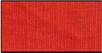	44.50 ± 0.99	53.30 ± 0.12	36.49 ± 0.58	64.59 ± 0.67	34.40 ± 0.22	17.67 ± 0.93
T4DR1	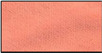	64.94 ± 0.82	36.41 ± 0.34	25.39 ± 1.0	44.39 ± 0.54	34.89 ± 0.38	2.51 ± 0.09
T5DR1	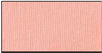	74.90 ± 0.08	23.19 ± 0.52	16.64 ± 0.20	28.54 ± 0.17	35.66 ± 0.76	0.81 ± 0.33
T6DR1	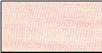	83.34 ± 0.12	10.32 ± 0.25	11.83 ± 0.33	15.70 ± 0.74	48.89 ± 0.26	0.25 ± 0.42

**Table 3:** Colour fastness values according to greyscale index.

Sample	Degradation					
	Wash	Rub		Iron		
		Dry	Wet	Dry	Humid	Wet
TDR1	4.4	4.3	4.3	4.1	3.7	3.6
T2DR1	4.3	4.2	4.4	4.2	3.9	4
T3DR1	4.6	4.4	4.4	4.4	4	4.1
T4DR1	4.6	4.3	4.4	4.3	4.1	4.1
T5DR1	4.7	4.5	4.6	4.4	4.2	4.3
T6DR1	4.7	4.7	4.5	4.8	4.7	4.6

**Table 4:** ANOVA for value by sample.

Source	Number of Squares	Gl	Medium Square	F-Ratio	*p*-Value
Between groups	1.34889	5	0.269778	5.77	0.0008
Intra-groups	1.40333	30	0.0467778		
Total (Corr.)	2.75222	35			

**Table 5:** ANOVA for value by test.

Source	Number of Squares	Gl	Medium Square	F-Ratio	*p*-Value
Between groups	0.711111	2	0.355556	5.75	0.0072
Intra-groups	2.04111	33	0.0618519		
Total (Corr.)	2.75222	35			

**Table 6:** ANOVA for value by conditions.

Source	Number of Squares	Gl	Medium Square	F-Ratio	*p*-Value
Between groups	0.3225	2	0.16125	2.25	0.1253
Intra-groups	1.93917	27	0.071821		
Total (Corr.)	2.26167	29			

**Table 7:** BET surface areas, pore volumes, and average pore sizes.

Sample	Surface Area (m^2^/g)	Pore Volume (cm^3^/g)	Average Pore Size (nm)
LDH	114.3	0.21	10.07
LDHC	239.6	0.37	18.7
HDR1	102.2	0.15	11.8

**Table 8:** Binding energy hydrotalcite and HDR1.

C1s							
Hydrotalcite				HDR1			
B.E.	FWHM eV	Area	%	B.E.	FWHM eV	Area	%
284.56	1.1	121,809.04	85.68	284.61	1.46	67,532	68.39
285.22	1.02	7726	5.44	285.8	1.44	17,364.67	17.58
285.99	1.33	2552.77	1.8	287.14	2	4679.13	4.75
288.55	1.55	10,054.6	7.08	289.26	1.55	9139.44	9.28
O1s							
Hydrotalcite				HDR1			
B.E.	FWHM eV	Area	%	B.E.	FWHM eV	Area	%
530.29	1.56	39,229.75	11.89	530.9	1.56	61,928.33	13.38
531.49	1.68	278,048.9	84.29	531.98	1.71	383,308.49	82.93
532.81	1.59	12,566.04	3.82	533.37	1.59	17,051.09	3.69
Al2p3							
Hydrotalcite				HDR1			
B.E.	FWHM eV	Area	%	B.E.	FWHM eV	Area	%
73.82	1.47	9347.56	100	74.41	1.41	10,071.94	100
Mg1s							
Hydrotalcite				HDR1			
B.E.	FWHM eV	Area	%	B.E.	FWHM eV	Area	%
1308.68	1.57	61,905.82	100	1303.82	1.51	141,429.97	95.2
				1305.03	1.44	7617.21	5.1
N1s							
Hydrotalcite				HDR1			
B.E.	FWHM eV	Area	%	B.E	FWHM eV	Area	%
-	-	-	-	399.34	1.94	10,913.85	86.34
				401.18	1.66	1730.72	13.66

**Table 9:** DOE chemical attacks HDR1.

	pH (Basic–Acid)	Temperature	Stirring	Detached Dye
Sample n^o^	HCl or NaOH	150 or 200 °C	0 or 1200 r.p.m.	%
1	acid	150	1	7.218%
2	acid	150	0	7.109%
3	acid	200	1	7.654%
4	acid	200	0	7.425%
5	basic	150	1	17.657%
6	basic	150	0	16.264%
7	basic	200	1	15.742%
8	basic	200	0	13.760%

## Data Availability

Not applicable.
